# Large Left Atrial Appendage Causing Cryptogenic Stroke

**DOI:** 10.7759/cureus.5991

**Published:** 2019-10-24

**Authors:** Michael Cinelli, Mahmoud Shadi, Sherif El-Hosseiny, Farshid Daneshvar, Frank Tamburrino

**Affiliations:** 1 Internal Medicine, Staten Island University Hospital, Staten Island, USA; 2 Internal Medicine, Rutgers New Jersey Medical School, Newark, USA; 3 Cardiology, Staten Island University Hospital/Northwell Health, Staten Island, USA; 4 Cardiology, Staten Island University Hospital, Staten Island, USA

**Keywords:** cryptogenic stroke, left atrial appendage, structural heart disease, congenital anomaly

## Abstract

Cryptogenic stroke is a subtype of ischemic stroke for which no identifiable cause is found after routine diagnostic evaluation. It accounts for roughly 25% of ischemic strokes. Structural cardiac abnormalities such as patent foramen ovale, atrial septal defect (ASD), and a large left atrial appendage (LAA) are commonly associated risk factors in patients with cryptogenic stroke. We report a case of a patient with a history of a surgically repaired ASD found to have a large LAA without identifiable thrombus on both transthoracic echocardiogram and transesophageal echocardiogram after presenting with an acute cryptogenic stroke in the absence of any arrhythmias. We aim to emphasize the importance of the LAA, particularly if large, in the pathogenesis and development of cryptogenic strokes. Additionally, we discuss the necessity for clearly defined guidelines on whether to start anticoagulation in these patients.

## Introduction

Cryptogenic stroke is a subtype of ischemic stroke for which no identifiable cause is found after routine diagnostic evaluation [1]. Approximately 25% of ischemic strokes are determined to be cryptogenic [2].

Structural cardiac abnormalities such as patent foramen ovale (PFO), atrial septal defect (ASD), and a large left atrial appendage (LAA), which are not common risk factors for stroke in the general population, are increasingly found in patients with cryptogenic stroke [3]. The LAA is considered to be a major location for the formation of left atrial thrombi in patients with atrial fibrillation. There is limited data on the use of LAA flow velocity and morphology to better predict ischemic stroke risk. Recently, embolic stroke has been associated with atrial dysfunction and atrial cardiopathy even in the absence of atrial fibrillation [4].

We report a case of a patient presenting with a cryptogenic stroke found to have a large LAA without an identifiable thrombus on diagnostic imaging and in the absence of any arrhythmias.

## Case presentation

**Figure 1:**
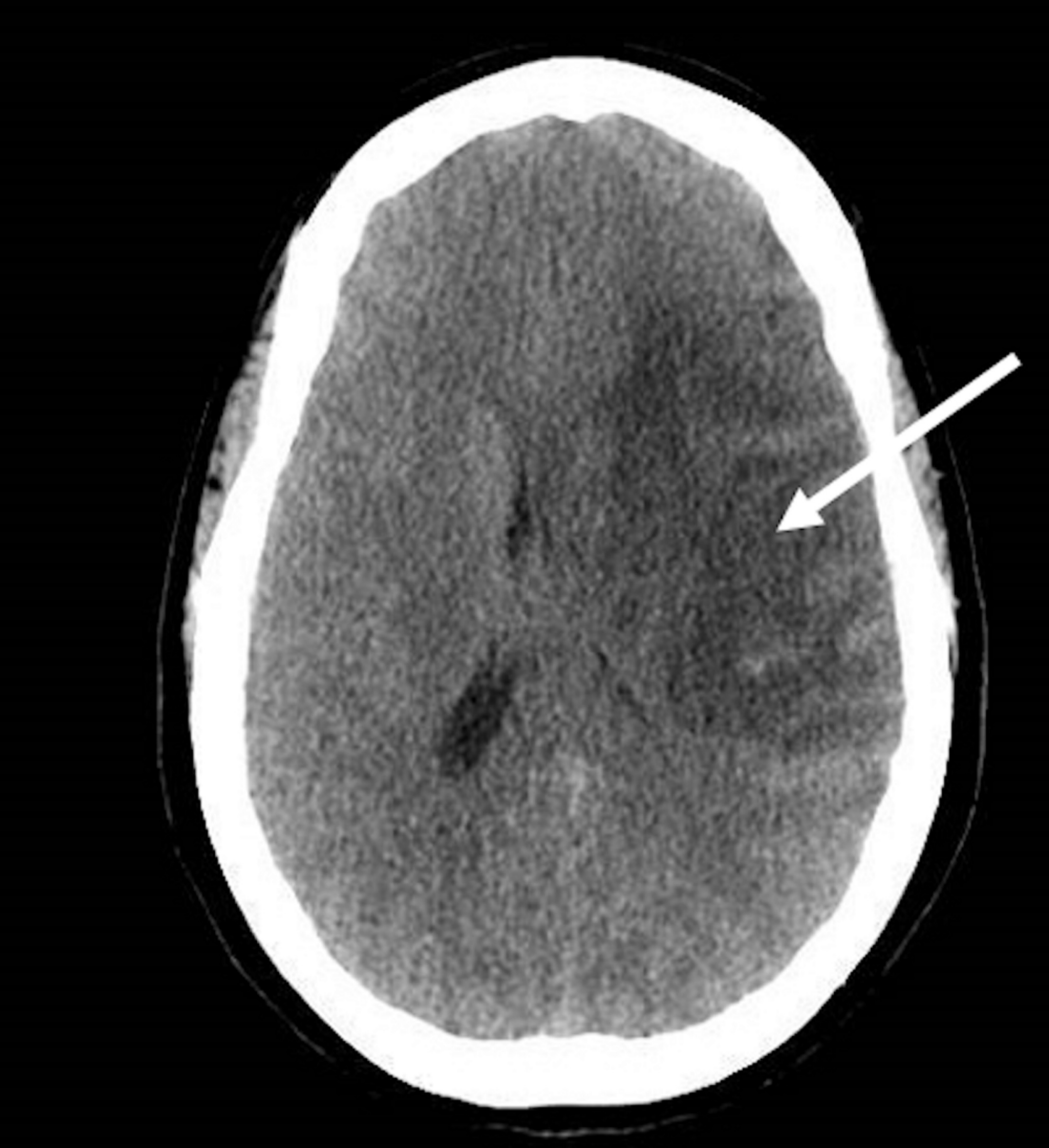
Computed tomography of the head showing a large acute on subacute infarction in the inferior portion of the left middle cerebral artery territory (white arrow).

**Figure 2:**
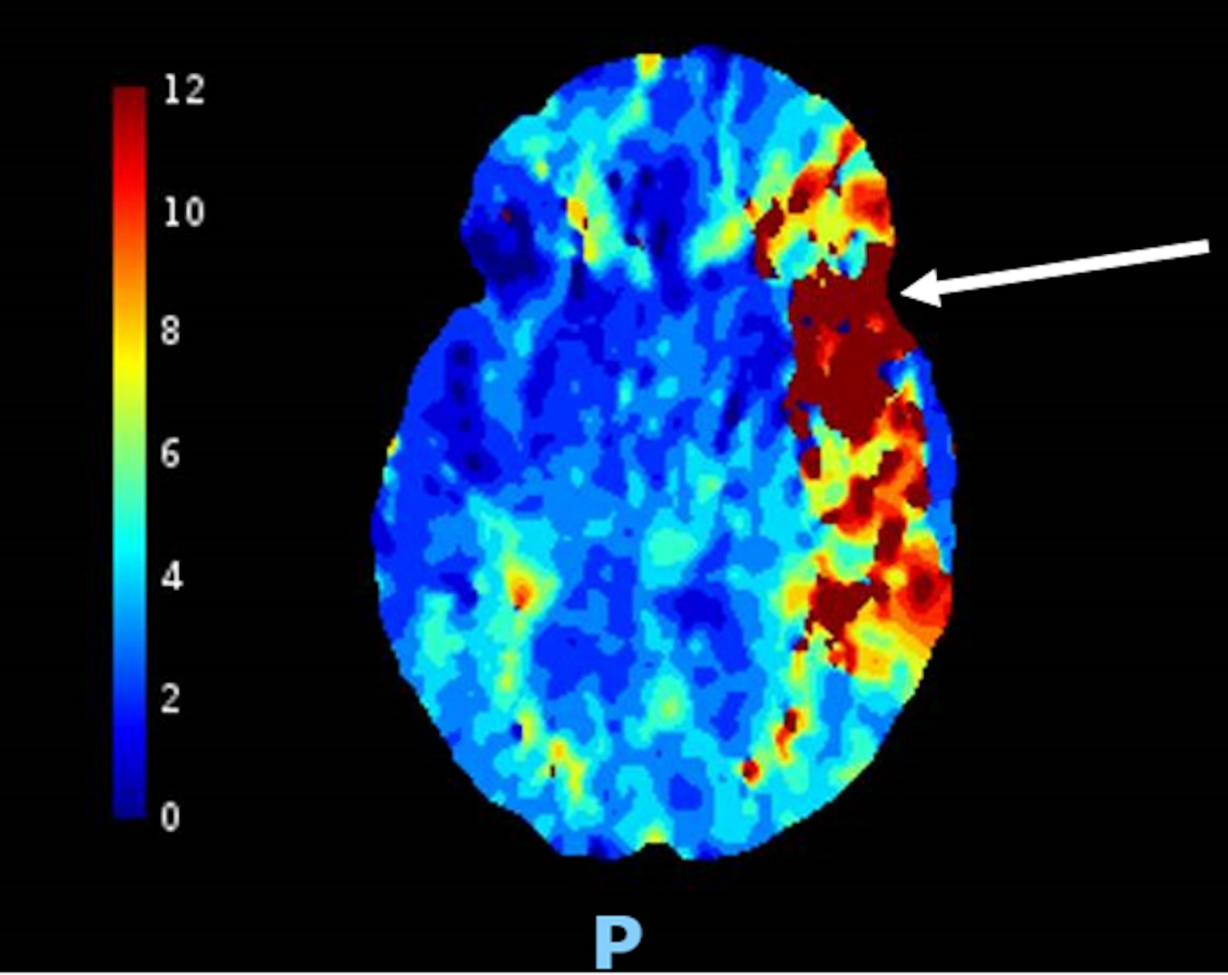
Computed tomography perfusion of the brain showing an acute core infarct with a large penumbra (red) in the inferior portion of the middle cerebral artery territory (white arrow).

A 54-year-old woman with a past medical history of hypertension and ASD surgically repaired at seven years of age presented to the emergency room after a fall and reported right sided weakness. The physical examination was notable for a left facial droop, aphasia, and 0/5 muscle strength in the right upper extremity as well as the right lower extremity. A stroke code was called, and the initial National Institutes of Health Stroke Scale (NIHSS) was 16. A computed tomography (CT) of the head showed a stable, large, acute or subacute infarction in the inferior portion of the left middle cerebral artery (MCA) territory with localized mass effect and no significant midline shift (Figure 1). A CT perfusion of the brain showed an acute core infarct of 70 mL, with a large penumbra of 17 mL (Figure 2). Neurology gave the patient one dose of tissue plasminogen activator. The decision was then made by the neurointerventional team to take the patient for mechanical thrombectomy. The patient underwent successful mechanical thrombectomy with partial re-cannulization of the left MCA. The patient tolerated the procedure well, and the NIHSS improved to 1 (for residual right lower extremity weakness).

**Figure 3:**
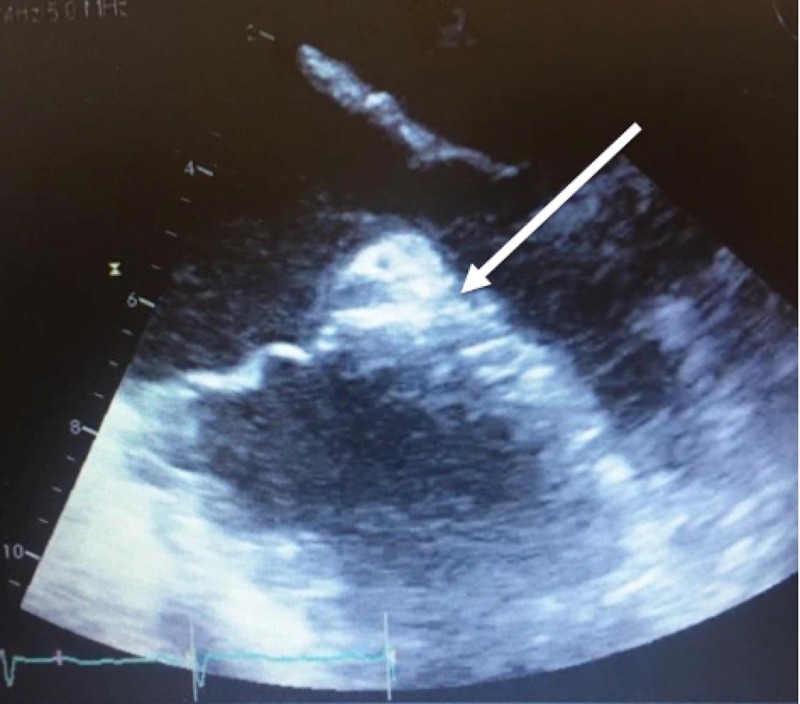
Transesophageal echocardiogram showing a large left atrial appendage (white arrow) without evidence of clot formation.

**Figure 4:**
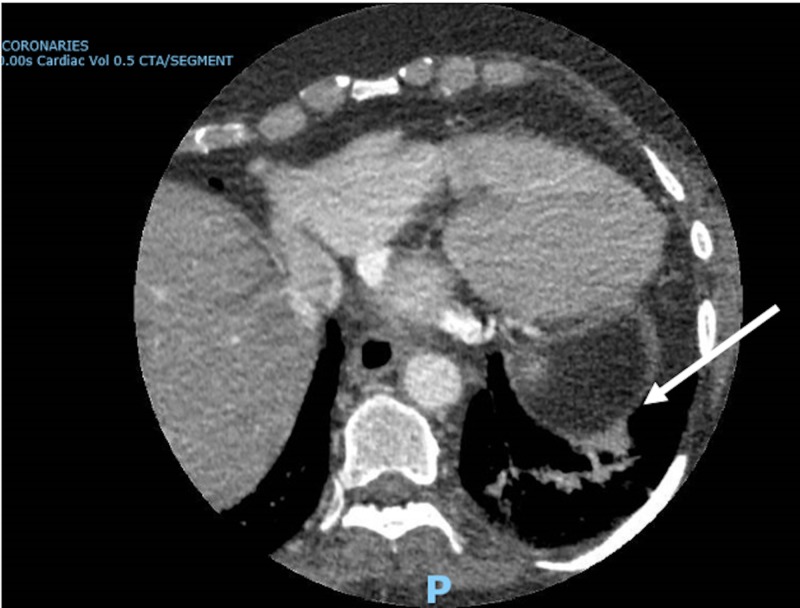
Computed tomography coronaries showing a large, multilobed left atrial appendage without evidence of thrombus (white arrow).

A transthoracic echocardiogram (TTE) showed a left ventricular (LV) ejection fraction of 60% to 65%, mildly increased LV wall thickness, normal LV internal cavity size, mild concentric LV hypertrophy, mild tricuspid regurgitation, and trace pulmonic valve regurgitation. An electrocardiogram showed normal sinus rhythm. Both neurology and cardiology recommended a transesophageal echocardiogram (TEE) for further evaluation due to the high clinical suspicion of thromboembolism. A TEE showed a large LAA without any evidence of clot formation (Figure 3, Video 1). Additionally, there was no evidence of PFO or ASD by color Doppler or agitated saline. Cardiology was concerned about the possibility of further clot formation and subsequent embolization from the large LAA due to the absence of any arrhythmic causes for ischemic stroke, particularly atrial fibrillation. A CT of the heart and coronaries showed mesocardia, normal size cardiac chambers and a large, multilobed LAA without any evidence of thrombus (Figure 4). The patient was subsequently started on aspirin 81 mg daily and apixaban 5 mg twice a day for anticoagulation and prevention of future ischemic stroke. The patient was discharged five days later to rehabilitation. Hypercoagulable work-up was done during outpatient follow-up and was negative.

**Video 1:** Transesophageal echocardiogram (TEE) showing a large left atrial appendage (LAA) without evidence of clot formation.

## Discussion

Cryptogenic stroke remains a major cause of morbidity and mortality. We present a case of a cryptogenic stroke in a patient found to have a large LAA with no thrombus formation in the absence of any arrhythmias, particularly atrial fibrillation or flutter. While no identifiable thrombus was found on TTE, TEE, or cardiac CT, the patient presented with acute ischemic stroke ultimately requiring neurointervention.

Associated risk factors for LAA-associated stroke include the LAA volume and morphology. A better understanding of the anatomy and physiology of LAA can provide valuable insight into elucidating the pathogenesis of these types of strokes. Multiple studies have shown statistically significant difference in the incidence of cryptogenic strokes in patients with different LAA morphologies although a clear association with a specific morphology remains unclear. Moreover, the effect of the LAA volume on the incidence of such strokes remains a relatively novel area of research and more studies are required to establish a definitive relationship. LAA volume has been found to be significantly higher in patients with cryptogenic stroke as well as transient ischemic attack [5]. Our patient demonstrates the potential complications that can arise from a patient with a large LAA. We hope to motivate practitioners to have a high index of suspicion for cryptogenic stroke in patients with a large LAA, even if there is no evidence of thrombus formation on routine imaging and if the patient is asymptomatic.

A large LAA has generally been considered a low risk for cardioembolic source of stroke and as such antithrombotic therapy indications have not been clearly defined [6]. Our patient eventually was started on anticoagulation in light of her acute ischemic stroke as well as associated risk factors. We aim to emphasize the need for clearly defined indications for anticoagulation in these types of patients. Fundamentally, the decision should always be determined on a case-by-case basis, considering patient risk factors, comorbidities and risks of adverse reactions to anticoagulation. However, more specific guidelines are needed to prevent these types of thromboembolic complications associated with the presence of this structural cardiac anomaly.

Finally, we aim to bring light to other potential risk factors for cryptogenic stroke such as structural heart disease not classically associated with thromboembolism. PFO has been commonly found to be implicated in patients with cryptogenic stroke as well as paradoxical emboli [3]. Our patient had a history of an ASD that was surgically repaired in childhood. We strive to highlight the importance of structural heart disease in patients with cryptogenic stroke and to be considered a risk factor to be kept in mind when dealing with patients with a known history of structural heart disease as well as those with surgical repair in the past. 

## Conclusions

Cryptogenic stroke has been associated with many risk factors in select patient populations such as those with structural heart disease. We present a patient with a history of surgically repaired ASD who was found to have a large LAA without identifiable thrombus on TTE or TEE after presenting with an acute cryptogenic stroke. We aim to emphasize the importance of the LAA, particularly if large, in the pathogenesis and development of cryptogenic strokes. Additionally, we discuss the necessity for clearly defined guidelines on whether to start anticoagulation in this patient population.
